# Proportion of adults in the general population of Stockholm County who want gender-affirming medical treatment

**DOI:** 10.1371/journal.pone.0204606

**Published:** 2018-10-05

**Authors:** Jill W. Åhs, Cecilia Dhejne, Cecilia Magnusson, Henrik Dal, Andreas Lundin, Stefan Arver, Christina Dalman, Kyriaki Kosidou

**Affiliations:** 1 Department of Public Health Sciences, Karolinska Institute, Stockholm, Sweden; 2 ANOVA, Center of Expertise in Andrology, Sexual Medicine, and Transgender Medicine, Karolinska University Hospital, Stockholm, Sweden; 3 Department of Medicine, Karolinska Institute, and Karolinska University Hospital, Stockholm, Sweden; 4 Centre for Epidemiology and Community Medicine, Stockholm County Council, Stockholm, Sweden; University of Westminster, UNITED KINGDOM

## Abstract

The number of patients presenting for care at gender clinics is increasing, yet the proportion of adults in the general population who want gender-affirming medical treatment remains essentially unknown. We measured the wish for cross-sex hormones or gender-affirming surgery, as well as other aspects of gender incongruence, among the general adult population of Stockholm County, Sweden. A population-representative sample of 50,157 Stockholm County residents ages 22 and older comprise the Stockholm Public Health Cohort. They were enrolled in 2002, 2006, and 2010 and followed-up in roughly 4-year intervals, with questions on health, lifestyle and social characteristics. In 2014, participants received the item “I would like hormones or surgery to be more like someone of a different sex.” Two additional items concerned other aspects of gender incongruence: “I feel like someone of a different sex”, and “I would like to live as or be treated as someone of a different sex.” Each item had four answer options (“Not at all correct”, “Somewhat or occasionally correct”, “Quite correct”, and “Absolutely correct”). For each item, any of the three affirmative answer choices were considered as some level of agreement. Calibration weights were used to estimate population-representative rates with 95% confidence intervals. The desire for cross-sex hormones or surgery was reported by 0.5% (95% CI, 0.4%–0.7%) of participants. Feeling like someone of a different sex was reported by 2.3% (95% CI, 2.1%–2.6%). Wanting to live as or be treated as a person of another sex was reported by 2.8% (95% CI, 2.4%–3.1%). These findings greatly exceed estimates of the number of patients receiving gender-affirming medical care. Clinicians must be prepared to recognize and care for patients experiencing discomfort due to gender incongruence and those who would like gender-affirming medical treatment.

## Introduction

Gender incongruence describes when one’s gender identity (inner sense of one’s own gender) differs from the sex assigned to the individual at birth. Some people experience distress due to gender incongruence. If the distress is severe, a person may meet the criteria for Gender Dysphoria, according to the fifth edition of the Diagnostic and Statistical Manual of Mental Disorders (DSM-5)[1], known as Transsexualism in the International Classification of Diseases (ICD-10)[2]. Individuals with gender dysphoria often express a need to alter the sex characteristics of the body. Some may seek gender-affirming medical care, including cross-hormone treatment and gender-affirmation surgery, in order to ease bodily dysphoria. Other options, such as socially transitioning to another gender, also play an important role in helping alleviate gender dysphoria for many.

There exist few estimates of the number of individuals in the population affected by gender dysphoria. Often, these estimates have been extrapolated from the numbers of patients who have been diagnosed clinically, or the number who are undergoing gender care or treatment in a specialist clinic. Two recent meta-analyses estimate the global prevalence of diagnosed gender dysphoria at 4.6 per 100,000[3] and 6.8 per 100,000[4]. The meta-analytical rate of gender-affirmation surgeries is estimated at 5.5 per 100,000[4]. Relying on solely clinical cases, though, underestimates the number of affected individuals[5], as many who desire gender-affirming medical treatment cannot, or do not, access this care.

Around the world, recent years have seen increasing numbers of patients presenting for care at gender clinics[3,6,7], and receiving transsexualism diagnoses. In Sweden, the National Board of Health and Welfare reports that about 1 in 100,000 was diagnosed with transsexualism in 2005, and in 2015 this number had risen to 8 per 100,000 (http://www.socialstyrelsen.se/nyheter/2017/konsdysforiokarsarskiltblandunga. Accessed August 3, 2018).

Individuals may continue to increasingly come forward seeking gender-affirming medical interventions. Estimation of the number of adults in the general population who want gender-affirming medical care is essential, so that health care systems and providers can be prepared.

To date, there is no direct estimation of how many adults in a general population want gender-affirming medical treatment. In one Dutch sexual health study, where participants were recruited from an internet panel, 1.1% of men and 0.8% of women experienced gender incongruence, and 0.9% of men and 0.3% of women wished to obtain gender-affirming hormones or surgery[8]. However, weaknesses of that study include a response rate of 20.9%, which may reflect non-response bias, and that the wish for gender-affirming medical treatment was only assessed among a sub-set of the study participants.

In the present study, among a large and well-characterized probability sample of the general population, we directly asked all study participants if they would like cross-sex hormones or gender-affirming surgery. We also surveyed for other aspects of gender incongruence among the random sample of 50,157 adults, ages 22 and older, across Stockholm County, Sweden.

## Materials and methods

### Setting

Stockholm County is the most populous county in Sweden, inhabited by a diverse population of 2 million individuals, 23% of whom are foreign born[9]. The county spans 2,500 square miles (~6,500 square km). About half of Stockholm County’s population lives within the Stockholm city limits and the rest resides in suburban, small town and rural locales.

### Study population

The Stockholm Public Health Cohort (SPHC)[10] is a population-based longitudinal cohort study with respondent recruitment occurring in successive waves (2002, 2006, 2010) and follow-up surveys taking place in roughly 4-year intervals (2007, 2010, 2014). The SPHC sampling frame consists of all adults in the Swedish Total Population Register and residing in one of Stockholm County’s 39 municipalities and urban districts. For each wave, an area-stratified random sample of approximately 50,000 adults aged 18–84 years (2002, 2006), or 18 years and older (2010) was invited to complete self-administered health questionnaires. The public health surveys included assessments of physical and mental health, lifestyle and socioeconomic characteristics. Data were collected using postal or web-based questionnaires in Swedish, English, Arabic and Polish, languages commonly spoken around Stockholm. The 2014 follow-up questionnaire also contained three items on gender incongruence. The full survey instruments are available online from the Stockholm County Council (http://folkhalsoguiden.se/halsa-stockholm/halsa-stockholm1, accessed August 22, 2017).

The current study population includes all participants in the SPHC recruited in 2002, 2006 and 2010 who responded to the 2014 follow-up questionnaire (N = 50,157). At the time of the 2014 follow-up survey, study participants were age 22 and above. Response rates to the initial surveys were 63%, 62%, and 56% in 2002, 2006, and 2010, respectively[10], and the retention rate of the combined 2002, 2006, and 2010 cohort to the 2014 follow-up survey was 71%[11]. Participants provided written informed consent and gave permission for record linkages to registries. Ethical approval was granted by the Stockholm Regional Ethical Review Board (Dnr:2010/1879-31/5; Dnr:2007/545-31).

### Gender incongruence and the wish for gender-affirming medical treatment

Items capturing aspects of gender incongruence, including the desire for gender-affirming medical interventions, were derived from the 27-item Gender Identity/Gender Dysphoria Questionnaire for Adolescents and Adults (GIDYQ-AA) based on items with factor loading exceeding 0.90[12] and in agreement with core elements of Gender Dysphoria in the DSM-5[1]. There are some individuals with gender dysphoria who identify as an alternative gender[1], transcending a binary male or female classification[13,14]. Therefore, the selected GIDYQ-AA items were adapted to enable identification with, or the desire to be, “a different sex” without requiring participants to indicate they feel like, or wish to be, a man or a woman. Finally, the measures were agreed upon for inclusion in the Stockholm Public Health Survey based on the statement of an expert panel from the Stockholm Gender Team, Karolinska University Hospital. (SA, CDh, KK).

All participants received three items related to gender incongruence: “I feel like someone of a different sex,” “I would like to live as or be treated as someone of a different sex,” and “I would like to change my body with hormones or surgery to be more like someone of a different sex”. Similar measures have been used previously in population-based surveys[8,15–17]. Each item had four answer options (“Not at all correct”, “Somewhat or occasionally correct”, “Quite correct”, and “Absolutely correct”). Individuals choosing any level of “correct” answer were considered to be in agreement with the item. Responses were further coded into two levels of agreement: “Somewhat or occasionally correct”, and “Quite or absolutely correct.” There were 644 individuals, (1.3% of participants), who did not respond to all three gender incongruence items, and 795, 921, and 921 individuals who did not respond to the 1^st^, 2^nd^, or 3^rd^ item above, respectively.

### Covariates

The 2012 Total Population Register provided data on sex (as assigned at birth or legally registered), age (categorized as 22–29, 30–44, 45–66, and 67+ years old) and country of birth (coded as Sweden, other European country, Asia, or rest of world). The Longitudinal Integration Database for Health Insurance and Labor Market Studies (LISA)[18] provided the data on educational attainment (9 years or less, secondary school, and university or post-secondary), and civil status as ever married (including same- and opposite-sex marriages, widows and separated or divorced) or never married.

### Statistical analysis

The prevalence of adults wanting gender-affirming medical care or agreeing with the other aspects of gender incongruence are reported using survey-weighted univariate statistics (in percentages and confidence intervals as measures of precision). Calibration weights were used to correct for the stratified random sample design as well as systematic non-response at baseline and follow-up[19]. Statistics Sweden provided the calibration weights based on available auxiliary variables from national registries, their association with selected survey variables, and association with probability of participating in the survey. The auxiliary variables included sex, age, country of birth, civil status, income, educational level, disability allowance and area of residence (sampling strata). A detailed description of the weights development is available from Statistics Sweden[20]. We estimated the proportions with 95% confidence intervals (based on standard error estimates calculated by Taylor series approximation). All analyses were performed using SAS software version 9.4 (SAS Institute Inc., Cary, NC, USA).

## Results

Characteristics of SPHC participants are described in Table 1. Approximately 0.5% (95% CI, 0.4%–0.7%) of the Stockholm adult population in 2014 agreed with the statement “I would like to change my body with hormones or surgery to be more like someone of a different sex” (Table 2). The wish for gender-affirming medical intervention was similar among males and females (0.6%, 95% CI, 0.4%–0.9%, and 0.4%, 95% CI,0.3%–0.5%, respectively).

**Table 1:** Characteristics of study participants. (N = 50 157).

Characteristics		n	%	Weighted %
	Total	50 157		
Registered Sex	Male	21 586	43.0	49.2
	Female	28 571	57.0	50.8
Age group	22–29 years	1 667	3.3	15.7
	30–44 years	9 332	18.6	30.3
	45–66 years	21 791	43.4	35.3
	67+ years	17 367	34.6	18.7
Country of birth	Sweden	43 091	85.9	73.4
	Rest of Europe	4 850	9.7	12.6
	Asia	1 289	2.6	8.4
	Rest of World	927	1.8	5.6
Education	< 9 years /unknown	6 226	12.4	17.0
	Upper secondary school	19 047	38.0	37.9
	University or Post-secondary	24 884	49.6	45.0
Civil status	Ever married	38 745	77.2	64.2
	Never married	11 412	22.8	35.8

**Table 2:** Proportion of adults who would like gender-affirming medical treatment, by sex and age. (N = 50 157).

I would like to change my body with hormones or surgery to be more like someone of a different sex.			
Age	Registered Sex	n	Weighted % (CI)
Total		121	0.5 (0.4–0.7)
	Male	61	0.6 (0.4–0.9)
	Female	60	0.4 (0.3–0.5)
22–29 years		16	1.0 (0.4–1.7)
30–44 years		24	0.4 (0.2–0.6)
45–66 years		57	0.6 (0.4–0.7)
67+ years		24	0.3 (0.1–0.4)

About 2.3% (95% CI, 2.1%–2.6%) of the population agreed with the item “I feel like someone of a different sex,” (Table 3). A similar proportion of women and men agreed with this measure (2.5% of women, 95% CI, 2.1%–2.8% and 2.1% of men, 95% CI, 1.7%–2.5%). The youngest age group was more likely than the oldest age group to agree with this item, with 4% (95% CI, 2.8%–5.2%) of participants ages 22–29 years feeling like someone of a different sex, and 1.1%, (95% CI, 0.9%–1.4%) of those ages 67+.

**Table 3:** Proportion of adults reporting other aspects of gender incongruence, by sex and age. (N = 50 157).

Age	Registered Sex	I feel like someone of a different sex.		I would like to live as or be treated as someone of a different sex.	
		n	Weighted % (CI)	n	Weighted % (CI)
Total		770	2.3 (2.1–2.6)	779	2.8 (2.4–3.1)
	Male	309	2.1 (1.7–2.5)	218	2.0 (1.6–2.5)
	Female	461	2.5 (2.1–2.8)	561	3.5 (3.0–3.9)
22–29 years		72	4.0 (2.8–5.2)	107	6.3 (4.7–7.9)
30–44 years		219	2.5 (2.1–3.0)	284	2.9 (2.5–3.4)
45–66 years		329	2.0 (1.7–2.3)	286	2.0 (1.7–2.3)
67+ years		150	1.1 (0.9–1.4)	102	1.0 (0.6–1.1)

There were 2.8% (95% CI, 2.4%–3.1%) of participants who agreed with the aspect of gender incongruence: “I would like to live as or be treated as someone of a different sex,” (Table 2). More females than males agreed with this item (3.5%, 95% CI, 3.0%–3.9% versus 2.0%, 95% CI, 1.6%–2.5%, respectively). Positive responses to this measure differed across age groups, with 6.3% of 22-29-year-olds (95% CI, 4.7%–7.9%), and 1.0% (95% CI, 0.6%–1.1%) of those ages 67+ agreeing with this statement. Across all three items, prevalence increased with lower age for both registered males and registered females.

Overall, 3.9% (95% CI 3.6%–4.3%) of individuals agreed with one or both measures, that they feel like someone of a different sex, and/or they wish to live as or be treated as someone of a different sex. Of those who feel like someone of a different sex, about half agreed that they wished to live as or be treated as someone of a different sex. Approximately 16% (95% CI, 11.8%–21.1%) of respondents who feel like a different sex, and 15% (95% CI, 11.0%–19.5%) of those who would like to live as or be treated as a different sex, wished to change their body with hormones or surgery. In other words, roughly 1 in 6 individuals experiencing either of these aspects of gender incongruence wished to pursue gender-affirming medical care.

When positive responses to the items were divided into two levels of agreement, “somewhat or occasionally correct” or “quite or absolutely correct,” the same proportion of males and females (0.2%, 95% CI, 0.1%–0.3%) answered with “quite or absolute” agreement that they would like to change their bodies with hormones or surgery to be more like someone of a different sex (Table 4).

**Table 4:** Level of agreement with aspects of gender incongruence, by sex. (N = 50 157).

	Somewhat or occasionally correct		Quite or absolutely correct	
	n	Weighted % (CI)	n	Weighted % (CI)
I feel like someone of a different sex.				
Male	172	1.3 (1.0–1.6)	137	0.9 (0.6–1.1)
Female	283	1.5 (1.2–1.8)	178	1.0 (0.7–1.2)
I would like to live as or be treated as someone of a different sex.				
Male	134	1.3 (0.9–1.7)	84	0.7 (0.5–1.0)
Female	434	2.5 (2.1–2.8)	127	1.0 (0.7–1.2)
I would like to change my body with hormones or surgery to be more like someone of a different sex.				
Male	41	0.5 (0.3–0.7)	20	0.2 (0.1–0.3)
Female	34	0.2 (0.1–0.3)	26	0.2 (0.1–0.3)

## Discussion

This is the first large-scale study measuring the wish for gender affirming medical care in a representative sample of the general population. Approximately 0.5% of adults in Stockholm County would like to change their body with hormones or surgery to be more like a person of a different sex. The proportion wanting hormones or surgery was similar among registered men and women. Other aspects of gender incongruence were more commonly reported: 2.3% of adults in Stockholm County agreed that they feel like someone of a different sex and 2.8% reported they would like to live as or be treated as someone of a different sex.

There are few representative population-based studies of gender incongruence conducted previously, and our survey is the first that enabled a random sample of adults in the general population to directly report if they would like cross-sex hormones or surgery. As well, unique to our study, the items in our survey were designed using non-binary gender terminology, so as not to exclude any individuals who may not necessarily identify as a man or as a woman.

In the Netherlands, a survey of an internet-panel assessed rates of psychologically experiencing oneself as a man or a woman[8], (opposite to the sex assigned at birth). Participants who identified equally or stronger with the opposite sex than with the sex assigned at birth were asked if they wanted hormones or surgery, numbering 0.9% of men and 0.3% of women.

A total of 3.9% of participants in our study agreed that they either feel like, or wish to live as or be treated as someone of another sex, or both. Our findings are similar to those in the Dutch study mentioned above[8], where 4.6% of men and 3.2% of women identified equally with both sexes, and 1.1% of men and 0.8% of women reported gender incongruence. In a representative population-based study from Flanders, Belgium, using similar measures as the Dutch study, 2.0% of individuals (95% CI, 1.4%–2.7%) identified equally as a man and a woman, while 0.6% (95% CI, 0.2%–1.0%) experienced gender incongruence[16]. In contrast, in a large-scale representative survey conducted across 19 US states, 0.5% of adults (95% CI 0.46%– 0.61%) identified themselves as transgender to a telephone interviewer[21].

The proportion of adults in our population-based study who would like gender-affirming medical interventions vastly exceeds estimates drawn from clinical cases. The meta-analytic prevalence for receipt of gender affirmation treatments globally is estimated to be 5.5 (95% CI, 0.5–10.5) per 100,000[4]. Individuals who are experiencing gender dysphoria and/or who are wishing to pursue gender-affirming medical treatment may not present in the clinic for a number of reasons, including social or financial constraints, feelings of shame, discomfort in the clinical setting, or lack of access to quality care[5,8,22].

Nevertheless, the number of individuals, particularly young, seeking care at gender clinics has increased sharply in Sweden and around the globe[3]. The reasons for these increases may include greater access to information about gender identity-related problems, increased awareness among referring providers, improved access to quality gender care, or greater acceptance of gender variance. As gender identity-related problems receive greater media attention, and society becomes more open, more individuals may seek gender-affirming medical treatment. Our findings indicate that far more persons than previously thought would like gender-affirming medical interventions, and health care systems and providers must be prepared to facilitate care for these individuals.

Though it has previously been implied that physicians will not meet large numbers of transsexuals in practice[23, 24], our findings suggest the average general practice physician with a panel size greater than 1,500[25] may indeed encounter a couple dozen patients with gender incongruence. Critically, though, providers in many settings report they need more training to appropriately care for gender incongruent persons[26–28].

The population-based survey design of the present study necessitates that some important considerations are kept in mind. Swedish society is reportedly tolerant toward gender variant individuals[29] and our results may not be readily generalizable to countries with a low tolerance towards gender diversity[3] or where gender-affirming treatment is not available or accessible[30]. While confidentiality is ensured for survey participants, some may hesitate to report aspects of gender incongruence, which would result in an underestimation. Additionally, it is unknown if gender incongruence is more or less common among survey non-responders. However, we used calibration weights to reduce the risk of such bias.

The survey items in this study were designed to capture some aspects of gender incongruence, as described in the DSM-5[1], including feeling like, wanting to live as, and wanting to be treated as someone of a different sex. These self-report measures may over-estimate feelings of gender incongruence by capturing individuals’ wishes to act like or be treated like a different sex, rather than feeling discomfort with one’s assigned sex or sexual characteristics. Particularly, regarding the item “I wish to live as or be treated as someone of a different sex”, this could have captured respondents’ desires for societal advantages of a different sex, or rejection of gender roles or gender stereotypes, rather than feelings of inappropriateness of one’s assigned sex. It is noteworthy, however, that a similar proportion of participants agreeing with the item “I feel like someone of a different sex” and the item “I would like to live as or be treated as someone of a different sex” express the wish for gender-affirming hormones or surgery (16% and 15%, respectively).

We must also consider that there are other aspects of gender incongruence that are not captured by the survey items, and thus, the study’s findings may underestimate the proportion of the population who experience gender incongruence. We found that individuals may align with some but not other aspects of gender incongruence that are assessed, similar to findings in the clinical setting[31].

Additionally, our data may or may not capture individuals who have undergone an earlier gender transition, or who wish to live as a different gender but not pursue cross-sex hormones or surgery. For these few individuals to be represented, or not, in our data, would minimally, if at all, affect the findings. In all, this study remains the most rigorous and largest epidemiological study to date, identifying the prevalence of adults who would like gender-affirming medical interventions in a representative sample of the general population.

## Conclusions

The prevalence of adults in Stockholm County who want gender-affirming medical interventions is far greater than previous clinic-based estimates imply. Individuals of all ages are affected by aspects of gender incongruence, especially young adults. Individuals wishing to pursue gender-affirming medical interventions may continue to increasingly present for care, and clinicians must be prepared to recognize and care for patients experiencing gender identity-related discomfort, as well as those who have received or would like gender-affirming medical treatment.
